# Application of Gellan Hydrogel and Kaz-6 in Wheat Seed Coating for Improved Productivity and Environmental Resilience

**DOI:** 10.3390/polym17101330

**Published:** 2025-05-14

**Authors:** Bagila Tursynova, Tolganay Zharkynbek, Rauash Mangazbayeva, Nurzhan Mukhamadiyev, Raushan Koizhaiganova, Gulnaz Mengdibayeva, Assel Ten, Bayana Yermukhambetova, Grigoriy Mun, Valentina Yu

**Affiliations:** 1 Laboratory of Synthetic and Natural Medicinal Compounds Chemistry, A.B. Bekturov Institute of Chemical Sciences, 106 Sh. Ualikhanov St., Almaty 050010, Kazakhstan; tursynova_0101@mail.ru (B.T.); k_raushan@yahoo.com (R.K.); assel.ten@gmail.com (A.T.); yuvkconst@gmail.com (V.Y.); 2 Faculty of Chemistry and Chemical Technology, Al-Farabi Kazakh National University, 71/23 Al-Farabi Ave., Almaty 050040, Kazakhstan; baya_yerm@mail.ru (B.Y.); mungrig@yandex.ru (G.M.); 3 Department of Chemical and Biochemical Engineering, Satbayev University, 22 Satbayev St., Almaty 050013, Kazakhstan; r.mangazbayeva@satbayev.university; 4 Department of Biological Plant Protection, Kazakh Research Institute of Plant Protection and Quarantine Named After Zh. Zhiyembayev, 1 Kultobe St., Almaty 050070, Kazakhstan; nurzhan-80@mail.ru (N.M.); www.gulnaz87.kz@mail.ru (G.M.)

**Keywords:** gellan, hydrogel, dimethyl[1-(2-ethoxyethyl)-4-hydroxypiperidin-4-yl]phosphonate (Kaz-6), coating, wheat, growth stimulant, drought

## Abstract

Drought is a major environmental constraint that negatively affects crop germination, seedling establishment, and overall yield. This study presents a sustainable approach to improving wheat productivity under water-deficit conditions through the application of a gellan gum-based hydrogel enriched with the growth stimulant. The hydrogel was synthesized by inducing ionic gelation of gellan gum using potassium chloride and ammonium sulfate, forming a robust, cross-linked polymer network. Wheat seeds were coated with one to eight layers of the hydrogel using a sequential dipping and drying process. Optimal seedling performance was achieved with a two-layer coating, balancing sufficient water retention with adequate gas exchange. FTIR spectroscopy and pH analysis confirmed ionic interactions between Kaz-6 and the carboxyl groups of gellan, supporting its stable incorporation within the polymer matrix. Mechanical characterization showed that ammonium sulfate significantly enhanced gel strength and cross-linking density compared to potassium chloride. Laboratory germination assays and greenhouse trials demonstrated that seeds coated with gellan hydrogel containing Kaz-6 showed enhanced germination rates, greater biomass accumulation, and significantly improved drought tolerance—surviving up to 10 days longer than controls under water-limited conditions. These findings highlight the potential of biopolymer-based hydrogels as eco-friendly seed coating materials that can improve crop resilience and productivity in arid environments. The proposed formulation aligns with sustainable agriculture goals and represents a promising direction for future field-scale applications in climate-adaptive farming systems.

## 1. Introduction

According to experts, the global demand for food, fiber, and bioenergy will significantly increase in the coming decades due to population growth and rising purchasing power in developing countries [1,2]. However, these predictions coincide with forecasts of potential global warming, which threatens agricultural lands with desertification and an increase in extreme weather events such as prolonged droughts [3]. This situation highlights the importance of agronomic techniques aimed at improving the productivity of agricultural crops in field conditions. One of the most effective methods is seed coating technology, which involves pre-sowing treatment of seeds by enveloping them with a protective nutrient layer containing various components beneficial for seed development. This approach also increases the seed size and gives it a uniform shape.

Seed coating is particularly effective for small and irregularly shaped seeds, as it simplifies their sowing process. The coating often contains nutrients, growth stimulants, and protective substances that enhance the seeds’ biological, chemical, and physiological characteristics, improving seedling viability, resilience to adverse environmental conditions, and overall yield [4]. The technology of coating seeds with a thin, permeable pesticide layer was first used for cereal crops in the 1930s [5]. In the 1960s, the concept of covering small seeds with a thicker layer to increase their size and facilitate planting in agricultural production was introduced [6]. Over time, various coating agents have been developed, containing pesticides, polymers, plant hormones, growth stimulants, minerals, and other protective and nutritional substances to safeguard plants from diseases, drought, and environmental stress [7,8].

At present, there is a growing global demand for coated seeds, particularly in developing countries where agriculture plays a leading role in the national economy [9]. Commonly used coating materials include clay, talc, and hydrophilic natural and synthetic polymers with added protective and growth-stimulating substances [10]. According to experts, one of the most promising materials for seed coating technology is polymer hydrogels, which are known for their unique ability to reversibly retain enormous amounts of water—up to several hundred grams of water per gram of dry polymer [11,12]. Although hydrogels have only recently been applied to seed coating technologies [13], they have traditionally been used as moisture-retaining additives in soil to improve the water regime and reduce the frequency of irrigation for perennial plants, ultimately enhancing seed germination and crop survival [12].

Unlike their direct application in agricultural soils, the use of hydrogels for seed coating to produce granulated seeds for large-scale planting, especially aerial sowing in arid regions, has proven highly effective [14,15]. The applied polymer hydrogel forms a moisture-retaining protective layer around the seeds, reducing water loss while increasing the weight of small seeds due to the coating. These properties can be particularly beneficial in countries with a significant portion of agricultural land in arid regions, where the average annual rainfall is only 150–200 mm [16]. Studies have shown that coatings containing water-absorbing polymers can significantly improve seed germination and seedling emergence under drought conditions [12].

Owing to their inherent capacity for water retention and controlled release of bioactive compounds, polymeric hydrogels have demonstrated considerable efficacy in enhancing seed germination, promoting crop yield, and increasing plant resilience under abiotic stress conditions. In seed pelleting technologies, polymeric hydrogels are broadly categorized into synthetic variants—such as polyacrylamide and acrylic acid—and natural biopolymers including starch, cellulose, alginate, gellan gum, and chitosan. While synthetic hydrogels exhibit superior mechanical strength and structural integrity, they are often limited by poor biodegradability. Conversely, natural hydrogels offer enhanced environmental compatibility and biodegradability, albeit with reduced mechanical robustness. The strategic integration of these classes facilitates the development of semi-synthetic hydrogel matrices with optimized physicochemical properties tailored for agricultural applications.

The formulation of the pelleting matrix exerts a critical influence on seed physiological performance. The incorporation of agrochemicals such as insecticides, fungicides, and phytohormonal growth promoters into the hydrogel matrix has been shown to improve germination rates and enhance seedling vigor. Nevertheless, it is essential to calibrate the coating thickness precisely, as excessive encapsulation may impede radicle emergence, particularly under suboptimal moisture availability [17,18].

The study by Amirkhani et al. [19] investigated the effects of seed pelleting using superabsorbent polymers (SAPs) on the germination dynamics and stand establishment efficiency of red clover (*Trifolium pratense* L.). Red clover seeds were coated with the following selected SAP formulations at 2% of their seed weight: cross-linked potassium polyacrylate (PAL), cross-linked polyacrylamide-based polymer (PAM), PAM with graphite (PAM + G), and Starch-g-2-Propenoic acid (potassium salt) (STR). The water absorbency of each SAP formulation was >200 g water/g of polymer; STR had the greatest absorbency, at 352 g water/g of polymer. A seed coating method was developed, resulting in the uniform application of SAP from seed to seed. All SAP coating treatments increased germination compared to the 0% SAP coating in controlled environment studies in the lab. Three field trials were conducted for each seed coating treatment, providing a range of climatic soil conditions. Within each field trial, the STR with the greatest water absorbency had a higher stand for treatments sown by broadcasting followed by raking to incorporate seeds. Collectively, the selected SAP seed coating improved field stands compared to the non-treated controls.

It is important to emphasize that the use of hydrogels derived from natural polymers for seed pelleting enables the production of environmentally safe, high-performance seeds encapsulated in a protective, moisture-retentive coating. This bio-based film readily undergoes biodegradation in the soil, thereby ensuring minimal ecological impact and posing no harm to surrounding flora or the broader environment.

The study [20] examined the effects of a biodegradable hydrogel seed coating on the early developmental stages of corn (*Zea mays* L.). The hydrogel employed in the study was based on potato starch chemically modified with succinate, exhibiting an equilibrium water absorption capacity of 260 g of distilled water per gram of hydrogel. The germination performance and subsequent seedling growth of both coated and uncoated seeds were assessed as functions of (1) the quantity of modified starch applied to the seed surface and (2) water availability. Under moisture conditions approximating 77% of the soil’s field capacity, the coated seeds demonstrated a markedly higher germination rate compared to their uncoated counterparts.

In the study of reference [21], a multilayer seed pelleting technology was developed using an alginate-based hydrogel coating enriched with macro- and micronutrients (NPK, Cu^2+^, Mn^2+^, Zn^2+^), along with amino acids derived from the alkaline hydrolysis of mealworm larvae. The optimal application dose of 70 mL per 100 g of seeds resulted in the formation of a uniform coating that enabled controlled nutrient release (≤10%) while achieving high bioavailability (up to 100%). Germination tests conducted on *Cucumis sativus* L. (cv. Cornichon de Paris) demonstrated a 50% increase in seedling fresh biomass and a fourfold enhancement in root length relative to untreated seeds.

Among naturally derived polymers considered as water-retaining agents for seed pelleting applications, gellan gum is undoubtedly one of the most promising candidates. First discovered in 1978 by CP Kelco (San Diego, CA, USA), gellan is a linear anionic heteropolysaccharide that stands out among other polysaccharides due to its ability to form gels at relatively low polymer concentrations (approximately 1–2 wt%). These gels typically form upon the cooling of hot aqueous solutions but can also be produced via ionic diffusion into cold gellan solutions. The gelation behavior of gellan in aqueous environments has been extensively reviewed in several key studies [22,23].

Although the precise gelation mechanism of gellan remains partially unresolved, it is widely accepted that gelation is initiated by a conformational shift in gellan macromolecules from disordered coil states to ordered double-helical structures upon cooling. This transition is significantly enhanced by the presence of cations, particularly divalent ones such as Ca^2+^ and Mg^2+^, which function as ionic bridges between carboxyl groups of adjacent polymer chains. While monovalent ions reduce electrostatic repulsion between chains, divalent ions confer greater mechanical stability and integrity to the gel matrix at lower concentrations.

According to [24] cooling in the presence of K^+^ or Ca^2+^ ions induces a conformational rearrangement from random coils to double helices, which subsequently aggregate into a three-dimensional gel network. These structural transformations were characterized by using temperature-dependent NMR spectroscopy, which revealed increased line broadening and chemical shift variations indicative of reduced chain mobility.

Further elucidation of the gellan gel structure was provided by [25], who employed two-dimensional NMR techniques (NOESY and ROESY) to detect spatial interactions among monomeric units, confirming interchain associations within the double-helix configuration. NMR spectroscopy has proven to be a powerful tool not only for the structural verification of gellan but also for monitoring its behavior in response to changes in ionic strength, pH, and temperature. It was demonstrated that increasing the ionic strength of the medium enhances the shielding of negative charges along the polysaccharide backbone, thereby promoting helix formation and increasing gel density. This effect is substantiated by the observed chemical shift in the carboxyl group signals from glucuronic acid residues in the ^13^C NMR spectra.

Overall, gellan exhibits significant potential as a seed pelleting component due to its intrinsic biopolymeric nature, exceptional water-retention capacity, compatibility with bioactive compounds, and environmental safety. Its application in seed coating technologies may enhance germination performance, improve plant tolerance to abiotic stressors, and contribute to increased crop productivity across a wide range of plant species.

In light of the foregoing, the present study investigates the efficacy of utilizing gellan in combination with the growth stimulant Kaz-6 (dimethyl[1-(2-ethoxyethyl)-4-hydroxypiperidin-4-yl]phosphonate) within a wheat seed pelleting system. Gellan, a naturally derived, water-soluble gel-forming polymer, is a linear anionic heteropolysaccharide composed of repeating tetrasaccharide units consisting of 1,3-β-D-glucose, 1,4-β-D-glucuronic acid, 1,4-β-D-glucose, and 1,4-α-L-rhamnose [26]. The growth stimulant Kaz-6, previously synthesized and characterized by the authors of [27], demonstrates potent plant growth-promoting activity. Its molecular structure was elucidated through X-ray diffraction analysis [28].

## 2. Materials and Methods

### 2.1. Preparation of Gellan-Based Hydrogel for Seed Coating

The hydrogel material was prepared using Gelzan™ CM (gellan gum, ≥98.5% active substance content) obtained from Zhejiang DSM Zhongken Biotechnology Co., Ltd**.** (Guangzhou, China) without further purification. The salts, ammonium sulfate and potassium chloride, were purchased in granular form from LLP “Chemistry and Technology” (Almaty, Kazakhstan) and used without additional processing.

The hydrogel was obtained by slowly adding a 3 mol/L aqueous salt solution (potassium chloride or ammonium sulfate) to a 1.0% aqueous solution of gellan gum while stirring at room temperature. Gelation occurred within 1–6 h, depending on the salt concentration and type. The resulting two-phase system consisted of the formed gellan gel (lower phase) and the salt solution above the gel (upper phase). The system was left undisturbed for a specified time, after which the lower and upper phases could be easily separated from each other.

After gel formation, the solution containing the gel was transferred onto a coarse-pore Schott glass filter No. 1 (Himlaborpribor, Klin, Russia) to separate the gel from the sol fraction.

The degree of swelling (α) was determined gravimetrically using the following formula:

$$\propto =\frac{m_{s}-m_{d}}{m_{d}}$$

where m_s_ is the mass of the equilibrium-swollen hydrogel sample, and m_d_ is the mass of the dry hydrogel sample.

Samples were dried in a forced-air oven at 105 °C until a constant weight was achieved, with accuracy to 0.0001 g using a Sartorius analytical balance (Göttingen, Germany). The swelling degree was calculated at various salt concentrations and times to understand the gel’s behavior under different conditions.

### 2.2. Measurement of Elastic Modulus of Obtained Hydrogels and Calculation of Polymer Network Structural Parameters

The elastic modulus (G) under uniaxial compression of cylindrical hydrogel samples in the initial deformation region (2–5%) and the stress at 60% strain (*) were determined using a universal testing machine “Instron” (Instron Ltd., High Wycombe, UK) at a compression rate of 1.36 × 10^−4^ m/s. The experimental data and the calculation of the elastic moduli of the polymer hydrogel samples were processed using the “Instron Ltd.” Bluehill^®^ Universal software. To obtain average values, no fewer than five samples were tested.

The structural parameters of the gels—M_s_ (number-average molecular weight of active chains between cross-links) and n_s_ (concentration of active chains per unit volume)—were calculated based on the measured elastic modulus values and the theory of rubber elasticity according to the following equation [29]:

$$M_{s}^{mech}=\frac{RT\rho {\left(V_{2.S}\right)}^{1/3}{\left(\frac{V_{u}}{V_{F}}\right)}^{2/3}}{G},$$

where ρ is the density of the dry polymer, V_u_ is the volume of the undeformed dry network, and V_F_ is the volume of the network at the point of formation, n_c_ = ρ/M_s_.

### 2.3. Seed Coating Process

The resulting gellan hydrogel was ground using a Bosch Concept 7200 electronic disperser (Gerlingen, Germany) to achieve a paste-like consistency. The prepared hydrogel mass was enriched with 0.01% (*w*/*w*) Kaz-6(dimethyl [1-(2-ethoxyethyl)-4-hydroxypiperidin-4-yl ]phosphonate), a growth stimulant.

Wheat seeds were coated with the prepared gellan hydrogel by immersing them in the gel, followed by constant agitation for 30–35 min to ensure uniform coverage. The coated seeds were dried at 35 °C for 50 min using a Hurakan HKN-DHD16M dehydrator (Guangdong, China). The resulting coated seeds were photographed to document the changes before and after drying.

### 2.4. Characterization of Gel Formation

The gel formation process was evaluated at various concentrations of potassium chloride and ammonium sulfate (0.02–0.12 mol/L). The time and extent of gel formation were documented visually and with photographs, showing differences in the opalescence and density of the formed gel layers. The kinetics of gel formation were further analyzed by measuring the mass (m, wt.%) and gel fraction (G, %) over time using the gravimetric method.

The mass m was calculated using the following equation:

$$m=\frac{m_{gel}}{m_{sol}}\times 100\%,$$

where m_gel_ is the mass of the gel formed within a given time interval, and m_sol_ is the mass of the initial aqueous gellan solution in which gelation occurs.

The gel fraction yield G was determined according to the following equation:

$$G=\frac{m_{net}}{m_{gellan}}\times 100\%$$

where m_net_ is the mass of the dry polymer network obtained after drying the formed gel, and m_gellan_ is the mass of gellan present in the aqueous solution undergoing gelation.

For comparative analysis, gels formed with ammonium sulfate were found to form faster and have a higher gel fraction than those with potassium chloride, which is consistent with previous studies showing the superior gelation effect of divalent cations [30,31].

### 2.5. Laboratory Germination Tests

Laboratory tests were conducted to evaluate the germination and growth performance of the coated seeds. Four experimental groups were created: Control 1: non-coated seeds; Control 2: non-coated seeds with Kaz-6; Control 3: seeds coated with gellan hydrogel without Kaz-6; Experimental Group: seeds coated with gellan hydrogel containing Kaz-6.

Germination tests were performed in Petri dishes at 25 °C with a 12 h light/dark cycle. The length of sprouts was recorded daily for 10 days.

### 2.6. Effect of Hydrogel Layer Thickness

To determine the optimal hydrogel layer thickness, seeds were coated with one to eight layers of hydrogel using a repeated dipping–drying method.

The thickness of the polymer coating applied to the seeds was measured using a micrometer MK-25 0–25 0.01 (Chelyabinsk Instrument Plant, Chelyabinsk, Russia) with a measurement accuracy of 0.001 mm. The coating thickness was calculated based on the difference in the transverse diameter of the seed before coating and after the application and drying of the gel layer.

### 2.7. Drought Resistance Testing

The drought tolerance of the seedlings was evaluated using a Jacobsen germination apparatus [32] under controlled conditions (25 °C day/17 °C night temperature, 60% relative humidity). The seedlings were watered to full capacity every 2 days. After 7 days of growth, healthy seedlings were selected and subjected to water stress by withholding water until the seedling weight decreased to 70% of its initial mass.

### 2.8. Greenhouse Trials

Greenhouse experiments were conducted on seedlings from both the experimental and control groups, as previously described in Section 2.5. The seedlings were planted in pots under hydroponic conditions and monitored for 10 days. Biometric data, including shoot length, root length, and biomass, were collected and compared across the control and experimental groups.

## 3. Results and Discussion

In this study, the gelation processes (ion-induced sol–gel phase transition) in aqueous gellan solutions were investigated under the influence of low-molecular-weight electrolytes to develop a method for using gellan-based hydrogels for wheat seed coating. Potassium chloride and ammonium sulfate were used as low-molecular-weight electrolytes, as these are well known, highly effective fertilizers in agricultural chemistry [33,34]. As noted above, several studies have demonstrated [31,35,36] that divalent cations are more effective than monovalent cations in gellan gel formation in aqueous solutions due to ion-induced sol–gel phase transitions. This is because divalent cations can form cross-linking bridges by binding to carboxyl groups, whereas monovalent cations induce aggregation by suppressing electrostatic repulsion [35]. However, the effect of the nature and valency of the anion in the salt on the gellan gelation process in aqueous solutions has either not been investigated or has received insufficient attention.

The experiments were conducted using 1.0 wt% aqueous solutions of gellan gum, to which aqueous solutions of potassium chloride and ammonium sulfate at specified concentrations were added. The resulting biphasic system consisted of a gellan gel (lower phase) and the salt solution above the gel (upper phase). The system was left undisturbed for a predetermined time, after which the upper and lower phases could be easily separated.

**Figure 1:**
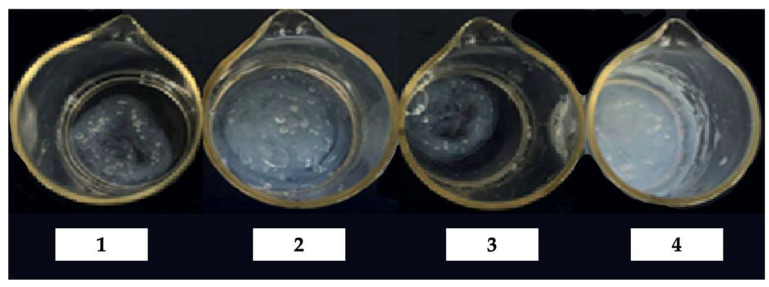
Gellan hydrogel formed after adding aqueous solution of potassium chloride (**1**,**2**) and of ammonium sulfate (**3**,**4**) to gellan solution. Salt concentration, mol/L: 0.02 (**1**,**3**); 0.08 (**2**,**4**). Gellan gum concentration: 1.0 wt%. Gel formation duration: 60 min.

It should be noted that gellan gel formation via ion-induced sol–gel phase transition is accompanied by the appearance of turbidity within the gel volume, with turbidity intensity increasing as more gel is formed. Figure 1 shows photographs of gellan gel formation after the addition of two different concentrations of potassium chloride and ammonium sulfate to the 1.0 wt% aqueous gellan solution. It is evident that the addition of ammonium sulfate results in significantly greater turbidity in the gellan–water system compared to potassium chloride. This suggests that ammonium sulfate more effectively induces the ion-induced sol–gel phase transition than potassium chloride.

It is important to note that ammonium sulfate contains only a monovalent cation, which is incapable of forming cross-linking bridges with carboxyl groups, unlike divalent cations [35]. Therefore, it is reasonable to conclude that the higher efficiency of ammonium sulfate in inducing gellan gel formation is due to the presence of a divalent sulfate anion and two ammonium cations, which together produce a significantly higher ionic strength in the aqueous solution compared to potassium chloride. Consequently, ionic strength plays a substantial role in the kinetics of gellan gel formation during the ion-induced sol–gel phase transition.

This leads to a decrease in the thermodynamic quality of the solvent, which is typically associated with a phase transition and the precipitation of the dissolved polymer. However, it is well-known that gellan macromolecules possess high rigidity, and during the phase transition, instead of forming a compact polymer precipitate, they generate a three-dimensional physical polymer network—i.e., a gel [24,25].

**Figure 2:**
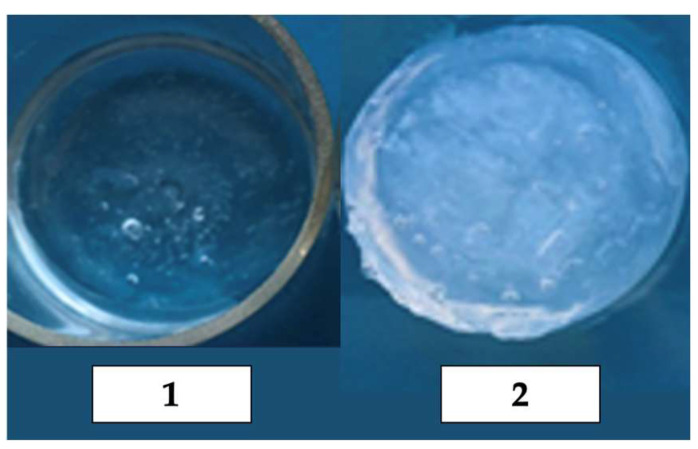
Gellan hydrogel formed after adding aqueous solution of ammonium sulfate to gellan solution. Ammonium sulfate concentration, mol/L: 0.04. Gellan gum concentration: 0.5 wt%. Gel formation duration, min: 30 (**1**); 360 (**2**).

Figure 2 presents photographs of gellan gels formed at two different time intervals after the addition of ammonium sulfate to a 0.5% aqueous gellan solution. It is evident that the turbidity of the gellan gel formed after 6 h is significantly higher than that of the gel formed after 30 min. This indicates that over time, gel yield increases along with turbidity, which is attributed to the formation of a more densely cross-linked polymer network.

As previously noted, the gellan gel formation process during the ion-induced sol–gel phase transition can be monitored visually by observing the increase in turbidity over time. This turbidity increase is evidently associated with the growing mass of the formed gel, the increasing gel fraction yield, and the progressive rise in the cross-linking density of the developing polymer network.

**Figure 3:**
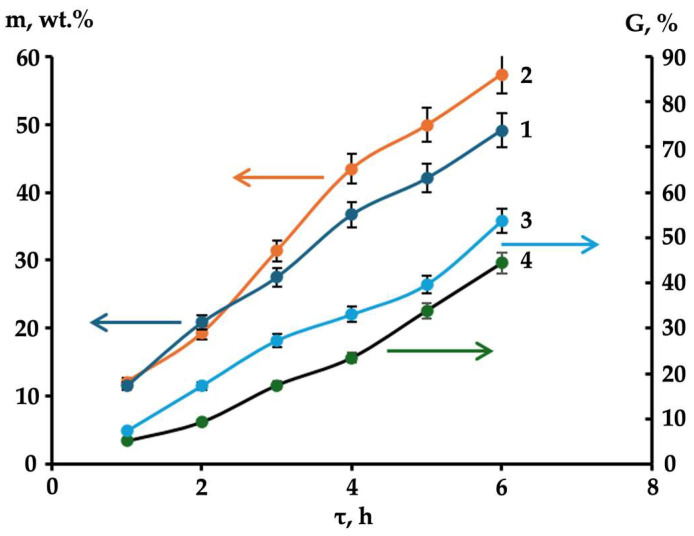
Kinetics of hydrogel mass formation (1,2) and gel fraction (3,4) in 1.0 wt% aqueous gellan solution at different concentrations of potassium chloride. Potassium chloride concentration in aqueous solution, mol/L: 0.08 (2,4) and 0.12 mol/L (1,3).

**Figure 4:**
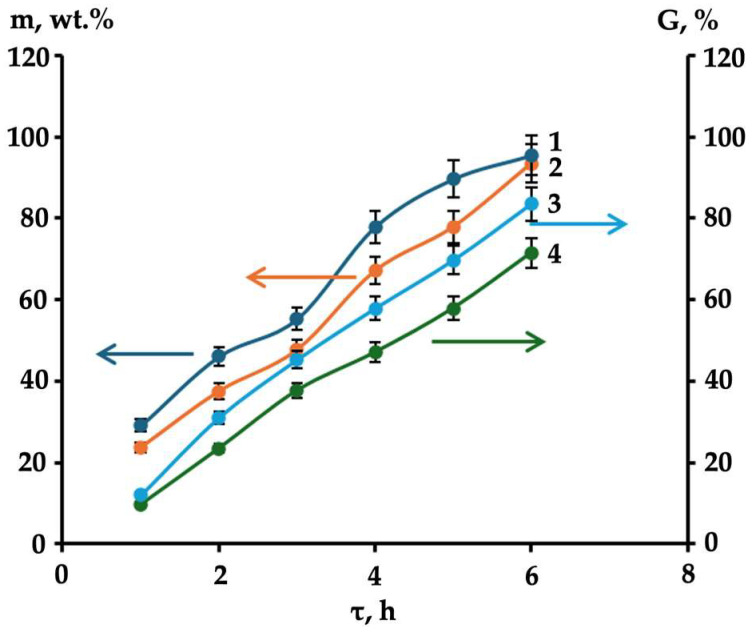
Kinetics of hydrogel mass formation (1,2) and gel fraction (3,4) in 1.0 wt% aqueous gellan solution at different concentrations of ammonium sulfate. Ammonium sulfate concentration in aqueous solution, mol/L: 0.08 (2,4) and 0.12 mol/L (1,3).

This is confirmed by the kinetics data for hydrogel formation (m) and the gel fraction (G) in a 1.0 wt% aqueous gellan solution with various concentrations of potassium chloride (Figure 3) and ammonium sulfate (Figure 4). It is evident that the mass of the formed gellan gel and the gel fraction yield increase when moving from the potassium chloride system (Figure 3) to the ammonium sulfate solutions (Figure 4).

The obtained data on the formation of gellan hydrogel under the influence of salts added to the gellan solution agree well with the findings of previous studies [31,35,36], which demonstrated that divalent cations are more effective than monovalent cations in gellan gel formation processes in aqueous solutions. This is because divalent cations can form cross-linking bridges by binding to carboxyl groups, whereas monovalent cations induce aggregation by suppressing electrostatic repulsion [35].

For gellan hydrogel samples formed under different gelation times (τ) and various concentrations of potassium chloride and ammonium sulfate, the equilibrium swelling degree in water (α) was determined. This value is expressed in grams of water per gram of dry polymer (g/g). The resulting data are presented in Table 1.

The data in Table 1 show that the obtained gellan hydrogels exhibit relatively high swelling degree (α) values, which are typical of polyelectrolyte hydrogels [12,13,15,19]. As the gelation time (τ) and the concentration of the added salt increase, the swelling degree of the formed gellan hydrogels decreases. This is likely due to the increased cross-linking density of the polymer networks caused by a higher number of physical cross-linking points, resulting from the reduced thermodynamic quality of the solvent and the prolonged formation time of the polymer network structure. Furthermore, hydrogels formed in gellan solutions in the presence of ammonium sulfate show lower equilibrium swelling degrees compared to those formed in the presence of potassium chloride. This observation can be attributed to the ability of ammonium sulfate to promote the formation of gellan gel networks with a higher degree of cross-linking than those formed in the presence of potassium chloride.

For the gellan hydrogels presented in Table 1, the elastic modulus G in the equilibrium swollen state was evaluated under uniaxial compression in the initial deformation range up to 5% (Table 2). Based on the obtained elastic modulus values, the structural parameters n_c_ (a parameter proportional to the cross-linking density of the polymer network, defined as the concentration of elastically active chains per unit volume) and M_c_ (the number-average molecular weight of active chains between cross-links) were calculated for the gellan hydrogels. The resulting data are presented in Table 3 and Table 4.

As can be seen from the data in Table 2, Table 3 and Table 4, an increase in the gelation time τ and the salt concentration leads to an increase in both the elastic modulus G and the value of n_c_, while the value of M_c_ decreases. This indicates an increase in the cross-linking density of the formed polymer network. Furthermore, the transition from potassium chloride to ammonium sulfate is also accompanied by an increase in n_c_ and a decrease in M_c_, indicating that the addition of ammonium sulfate to the aqueous gellan solution promotes the formation of a gel with a higher cross-linking density compared to the addition of potassium chloride.

As mentioned earlier, one of the most effective methods for improving grain yield and protecting seedlings from adverse external factors, such as water scarcity, is the preparation of seed material through seed coating with a polymer hydrogel. An essential feature of this process is the possibility of incorporating various nutrients into the protective hydrogel shell of the coated seeds, such as mineral fertilizers and plant growth stimulants, to support plant development during both seed germination and the vegetation period.

In this study, the material used for wheat seed coating was a hydrogel formed over 6 h by introducing ammonium sulfate and potassium chloride into an aqueous gellan solution at an equimolar ratio, with a total concentration of 0.12 mol/L. This is due to the fact that, under these conditions, the maximum yield of the formed gel is observed.

The resulting gellan hydrogel was ground to achieve a paste-like consistency (gel mass).

**Figure 5:**
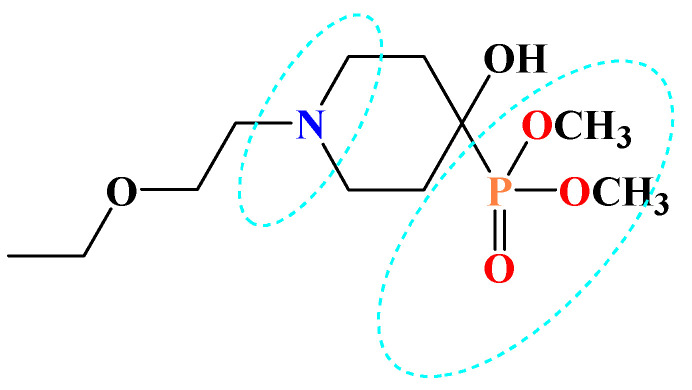
Structure of dimethyl[1-(2-ethoxyethyl)-4-hydroxypiperidin-4-yl]phosphonate.

A 0.01% (wt.%) solution of the previously developed derivative of ketopiperidine, dimethyl[1-(2-ethoxyethyl)-4-hydroxypiperidin-4-yl]phosphonate (Kaz-6), which exhibits pronounced plant growth-stimulating properties, was introduced into the resulting hydrogel-like mass. As established earlier, Kaz-6 at a concentration of 10^−3^% stimulates plant growth due to the presence of an amino fragment and a phosphonate group in the molecule (Figure 5) [37,38].

**Scheme 1:**
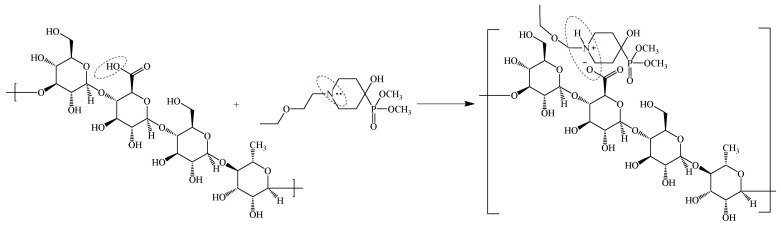
Interactions of Kaz-6 with gellan in aqueous solution and within gellan hydrogel matrix.

Considering the physicochemical properties, structural, and functional characteristics of gellan, it can be assumed that this natural polysaccharide is a promising polymer carrier for the plant growth stimulator Kaz-6, since it contains, among other things, free carboxyl groups that can interact with nitrogenous bases in aqueous solutions. As a result of such interaction, a salt complex may form, stabilized by ionic bonds between the nitrogenous functional groups of Kaz-6 and the carboxyl groups, in accordance with the proposed hypothetical complexation in Scheme 1.

As can be seen from the proposed scheme, the interaction between Kaz-6 and gellan, involving the nitrogenous functional groups of Kaz-6 and the carboxyl groups of gellan, is accompanied by the formation of corresponding ionic bonds, with the release of a proton into the surrounding aqueous medium. This is supported by the data on the pH changes (Metrohm 827 pH lab, Herisau, Switzerland) of the aqueous solution of Kaz-6 (10^−3^ mol/L) upon transitioning to the solution of the Kaz-6 (10^−3^ mol/L) and gellan mixture (Table 5).

**Figure 6:**
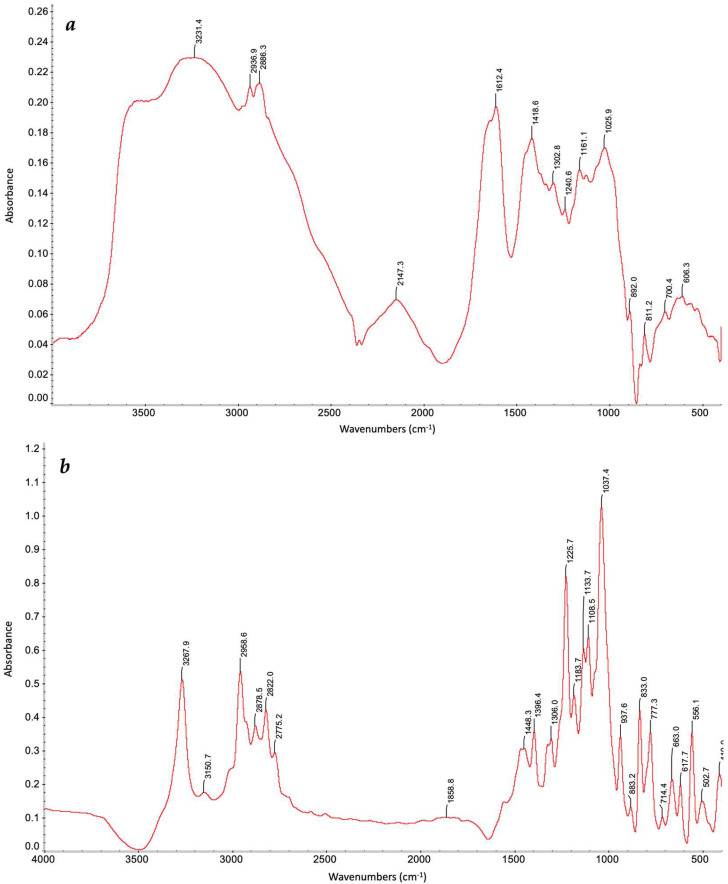

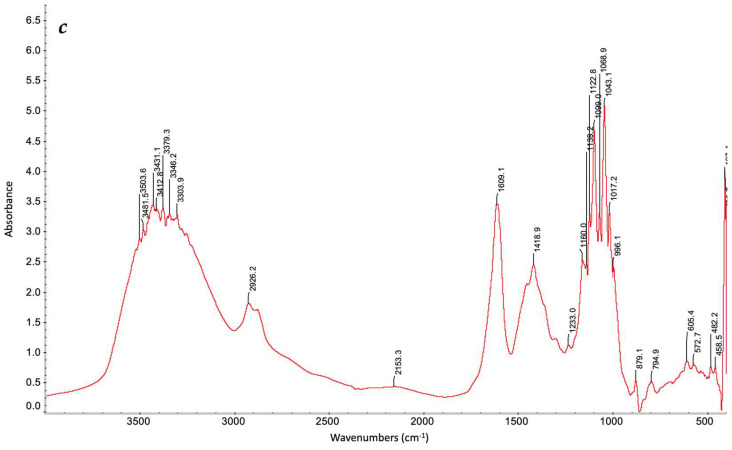
FTIR spectra of gellan (**a**), Kaz-6 (**b**), and Gellan + Kaz-6 (10^−3^ %) system (**c**).

In addition, an analysis of the FTIR spectra (Nicolet-5700 spectrometer, Thermo Fisher Scientific, Waltham, MA, USA) of gellan, Kaz-6, and the Gellan + Kaz-6 (10^−3^%) system was conducted (Figure 6).

As expected, the absorption regions of the C-H, O-H, N^+^-H (2750–3500 cm^−1^) and C-O, P-O (1000–1200 cm^−1^) bonds are complex and difficult to assign precisely. However, the absorption bands at 1612.4 cm^−1^ (broad) and 1418.6 cm^−1^ in the gellan spectrum are clearly attributed to the carboxyl group. In the Gellan + Kaz-6 spectrum, a shift in the C=O carboxyl fragment is observed to 1609.1 cm^−1^ (a 3.7 cm^−1^ shift to the lower wavenumber region), indicating the formation of an ionic bond between the carboxyl fragment of gellan and the nitrogenous functional groups of Kaz-6. The characteristic absorption band of the P=O bond at 1255.5 cm^−1^ in the oxophosphonate group of Kaz-6 is also observed in the Gellan + Kaz-6 spectrum at 1233.0 cm^−1^ (the small size of the band is explained by the low concentration of Kaz-6 in gellan, which is 10^−3^ mol/L).

**Figure 7:**
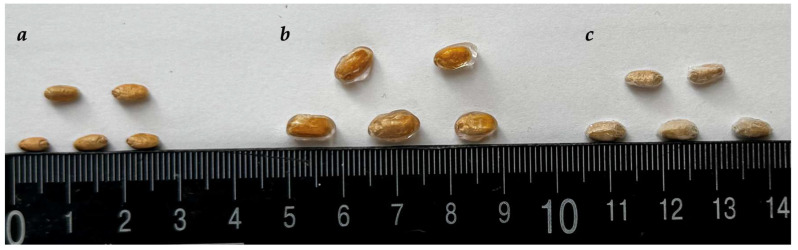
Wheat seeds without coating (**a**) and coated with gellan hydrogel before (**b**) and after drying (**c**).

The seed coating process is described in Section 2.3 “Materials and Methods”. Figure 7 illustrates the appearance of spring wheat seeds without coating (**a**) and coated with gellan hydrogel before (**b**) and after drying in the dehydrator (**c**).

Laboratory germination and seedling development tests were conducted on spring wheat seeds coated with gellan hydrogel containing the growth stimulant Kaz-6, using Petri dishes. The data obtained are presented in Table 6.

It was observed that the coated seeds began germinating on the 4th or 5th day and exhibited more intense seedling development compared to the untreated seeds (Control 1 and Control 2). The most pronounced positive effect was seen in seeds coated with gellan hydrogel containing Kaz-6, where seedling growth was significantly faster compared to the control groups.

In addition, the data in Table 6 indicate that Kaz-6 positively influences seedling development (Control 2), promoting faster growth compared to samples without Kaz-6 (Control 1). Moreover, a comparison between coated samples without Kaz-6 (Control 3) and untreated seeds (Control 1) shows that the presence of the gellan hydrogel shell enhances seedling development, even compared to untreated seeds with Kaz-6 (Control 2).

Statistical analysis of the group differences in growth measurements over the observation period (Days 2–10) was performed using one-way ANOVA. On Day 2, no statistically significant differences were observed among the groups (F(3,8) = 1.17, *p* = 0.3782). Starting from Day 3, significant differences became apparent, with ANOVA showing a progressive increase in F-values over time. For instance, on Day 4, ANOVA revealed a significant difference between the groups (F(3,8) = 108.54, *p* < 0.0001), and this trend continued through Day 10 (F(3,8) = 213.26, *p* < 0.0001). These results indicate that the application of gellan coating containing Kaz-6 significantly altered the measured parameter compared to the controls, especially in the later days of the experiment.

The data obtained suggest that increasing the thickness of the protective hydrogel layer on coated seeds may enhance the positive effect of the coating on seedling development. The layer thickness can be adjusted by applying multiple layers of hydrogel through repeated cycles of gel application and drying.

However, when studying the effect of the number of hydrogel layers on the germination of coated seeds, it was found that seed germination decreases as the number of gel layers increases. This is likely due to the thicker polymer shell hindering the penetration of wheat sprouts. It was determined that the optimal number of gel layers is two (Table 7), as further increasing the number of layers leads to a sharp decline in germination, eventually resulting in a complete cessation of seed germination.

As seen from the data in Table 7, the double coating of seeds with gellan hydrogel followed by drying after each layer application of the gellan hydrogel containing Kaz-6 provides the most active seedling development while maintaining a high seed germination rate (90–100%). Indeed, by the 10th day after planting, the height of seedlings from seeds coated with two layers of gellan was more than six times greater than that of seedlings from untreated control seeds and more than 1.5 times greater than that of seedlings from seeds coated with a single layer of gellan.

The seeds coated in this manner were planted in soil-filled pots (greenhouse conditions) under hydroponic cultivation, and biometric indicators of the seedlings were monitored for 10 days in comparison with the control and Kaz-6 treatments. As shown in Table 8, the seedlings from gellan-coated seeds containing Kaz-6 exhibited significantly more vigorous growth compared to the control samples of uncoated seeds with Kaz-6. The key growth parameters with their standard deviations for the germinated plants, the photographs of which are presented in Table 8, are provided above in Table 6.

As is well known, drought is one of the main factors affecting plant growth, survival, and productivity. The tested samples of gellan-coated seeds containing Kaz-6 were studied to identify signs that could predict the seedlings’ (sprouts) sensitivity or resistance to drought compared to the control (uncoated seeds in the presence of Kaz-6).

As an experimental sample, we selected wheat seed coating with a double layer of gellan hydrogel containing Kaz-6 and seed treatment with Kaz-6 without coating (as a control) before planting, to stimulate seedling growth. The hydrogel provides sufficient moisture for seed germination, while Kaz-6, containing nitrogen and phosphorus compounds (mineral nutrition), promotes better seed germination and seedling growth.

Thus, the wheat seed samples were placed in a root germinator to evaluate the root system of wheat. The seedlings were grown according to standard procedures.

After 7 days of growth, healthy plants of similar size were selected from each seed source (control and Gellan + Kaz-6 treatments), and watering was reduced until the seedling mass (plants and containers) decreased to 70% of its initial weight (to the pre-dawn moisture level, which was 50% of the turgor loss point), which helped avoid catastrophic cavitation of the xylem and harmful associated effects. After that, the seedlings were not watered.

**Figure 8:**
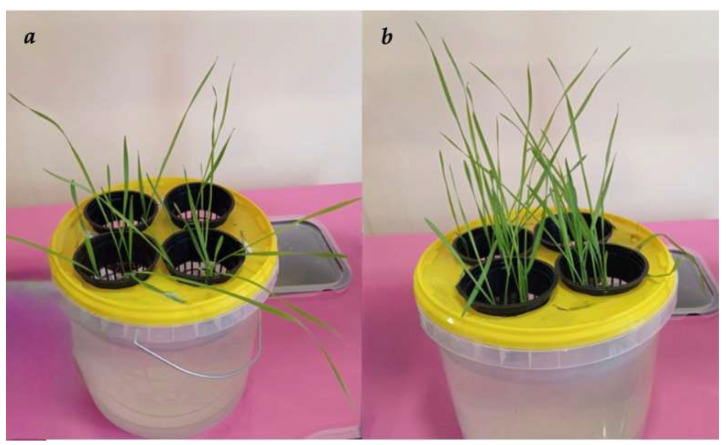
Seedlings in special Jacobsen apparatus, germinated from wheat seeds treated with Kaz-6 without coating (**a**) and from seeds coated with double layer of gellan hydrogel with Kaz-6 (**b**), under water deficit conditions (5th day after watering cessation).

Figure 8 presents photographs of seedlings in a special Jacobsen apparatus, germinated from the control wheat seeds treated with Kaz-6 without coating (a) and from seeds coated with a double layer of gellan hydrogel with Kaz-6 (b), under water deficit conditions (5th day after watering cessation).

It is evident that the seedlings in the experiment with gellan-coated seeds containing Kaz-6 demonstrate higher drought resistance, as reflected in the significantly more intense growth of green mass due to a more developed root system. Overall, it was found that seedlings from seeds coated with gellan hydrogel with Kaz-6 maintained viability under water deficit conditions for 10 days longer compared to the control samples. This suggests that the gellan hydrogel coating can act as a moisture-retaining barrier while simultaneously providing nutrient delivery and stimulating growth.

## 4. Conclusions

The findings of this study confirm the significant potential of gellan hydrogel combined with Kaz-6 for wheat seed coating. The coated seeds exhibited improved seedling growth, higher germination rates, and enhanced drought tolerance compared to uncoated seeds. The optimal number of hydrogel layers was found to be two, ensuring protection while maintaining oxygen and water permeability. Seedlings from gellan-coated seeds with Kaz-6 demonstrated prolonged survival under water deficit conditions, drying out 10 days later than the control samples. This indicates that gellan hydrogel coating can act as a moisture-retaining barrier while providing essential nutrients and growth stimulation.

These results suggest that gellan hydrogel with Kaz-6 is a practical and eco-friendly solution for improving wheat crop yield and resilience in arid regions. Future work should focus on field trials to further evaluate the scalability of this method and its long-term impact on agricultural productivity under varying environmental conditions.

## Figures and Tables

**Table 1:** Equilibrium swelling degree in water for gellan hydrogel formed by adding various concentrations of potassium chloride (KCl) and ammonium sulfate (AS) to gellan solution at different gelation times (g/g).

Gel Formation Time (τ), h	Equilibrium Swelling Degree in Water (α) of the Gellan Hydrogel Formed Upon Salt Addition to the Aqueous Gellan Solution, g/g			
	Potassium Chloride Concentration 0.08 mol/L	Potassium Chloride Concentration 0.12 mol/L	Ammonium Sulfate Concentration 0.08 mol/L	Ammonium Sulfate Concentration 0.12 mol/L
1.0	275.7 ± 5.5	226.9 ± 5.3	209.5 ± 4.3	199.1 ± 3.3
2.0	247.4 ± 3.3	216.9 ± 3.1	197.7 ± 2.9	175.3 ± 2.9
3.0	212.5 ± 3.1	193.1 ± 2.9	167.1 ± 2.9	147.9 ± 2.7
4.0	183.9 ± 2.9	173.7 ± 2.9	137.9 ± 2.7	129.7 ± 2.5
6.0	147.4 ± 2.9	117.7 ± 2.5	107.5 ± 2.6	93.5 ± 2.5

*statistically significant difference (p < 0.05).*

**Table 2:** Elastic modulus (G) of gellan hydrogels formed upon addition of various concentrations of potassium chloride and ammonium sulfate to aqueous gellan solutions at different gelation times.

Gel Formation Time (τ), h	Elastic Modulus (G) of Gellan Hydrogels Formed Upon Salt Addition to Aqueous Gellan Solution, kPa			
	Potassium Chloride Concentration 0.08 mol/L	Potassium Chloride Concentration 0.12 mol/L	Ammonium Sulfate Concentration 0.08 mol/L	Ammonium Sulfate Concentration 0.12 mol/L
1.0	0.75 ± 0.25	0.95 ± 0.15	1.11 ± 0.25	1.31 ± 0.25
1.0	0.91 ± 0.15	1.31 ± 0.25	1.51 ± 0.21	1.71 ± 0.21
3.0	1.71 ± 0.21	1.83 ± 0.35	2.73 ± 0.35	3.93 ± 0.35

**Table 3:** Values of cross-linking density parameter (n_c_) for gellan hydrogels formed upon addition of various salt concentrations to aqueous gellan solutions at different gelation times.

Gel Formation Time (τ), h	Values of the Cross-Linking Density Parameter (n_c_) of Gellan Hydrogel Formed Upon Salt Addition to Aqueous Gellan Solution, mol/m^3^			
	Potassium Chloride Concentration 0.08 mol/L	Potassium Chloride Concentration 0.12 mol/L	Ammonium Sulfate Concentration 0.08 mol/L	Ammonium Sulfate Concentration 0.12 mol/L
1.0	0.302 ± 0.101	0.383 ± 0.061	0.448 ± 0.101	0.528 ± 0.101
3.0	0.367 ± 0.061	0.529 ± 0.101	0.609 ± 0.085	0.690 ± 0.085
6.0	0.690 ± 0.085	0.739 ± 0.141	1.102 ± 0.141	1.586 ± 0.141

**Table 4:** Values of structural parameter M_c_ for gellan hydrogels formed upon addition of various concentrations of potassium chloride and ammonium sulfate to aqueous gellan solutions at different gelation times, g/mol.

Gel Formation Time (τ), h	Values of the Structural Parameter M_c_ of Gellan Hydrogel Formed Upon Salt Addition to Aqueous Gellan Solution, g/mol			
	Potassium Chloride Concentration 0.08 mol/L	Potassium Chloride Concentration 0.12 mol/L	Ammonium Sulfate Concentration 0.08 mol/L	Ammonium Sulfate Concentration 0.12 mol/L
1.0	2973 ± 991	2347 ± 371	2008 ± 452	1702 ± 325
3.0	2450 ± 404	1702 ± 325	1477 ± 205	1304 ± 160
6.0	1304 ± 160	1218 ± 233	817 ± 105	567 ± 51

**Table 5:** pH values of gellan, Kaz-6, and gellan with Kaz-6 solutions.

Solution	pH	Temperature
Gellan	6.38	24.3 °C
Kaz-6 (10^−3^ %)	8.48	
Gellan with Kaz-6	7.68	

**Table 6:** Comparison of wheat seedling growth length under different treatment conditions over 10 days.

Observation Day	No Coating (Control 1)	No Coating in the Presence of Kaz-6 (Control 2)	Coated with Gellan Without Kaz-6 (Control 3)	Coated with Gellan Containing Kaz-6	ANOVA Results for Data of Comparison of Wheat Seedling Growth Length Under Different Treatment Conditions Over 10 Days	
					F-Value	*p*-Value
1st	0.0 ± 0.00 cm	0.0 ± 0.00 cm	0.0 ± 0.00 cm	0.0 ± 0.00 cm		
2nd	0.0 ± 0.00 cm	0.1 ± 0.06 cm *	0.0 ± 0.00 cm	0.0 ± 0.00 cm	8.36	0.0075
3rd	0.0 ± 0.00 cm	0.2 ± 0.08 cm	0.0 ± 0.00 cm	0.0 ± 0.00 cm	108.54	<0.0001
4th	0.2 ± 0.09 cm	0.4 ± 0.07 cm	0.4 ± 0.18 cm	2.4 ± 0.21 cm *	136.73	<0.0001
5th	0.4 ± 0.18 cm	0.9 ± 0.29 cm	0.8 ± 0.05 cm	4.0 ± 0.38 cm *	234.73	<0.0001
6th	0.6 ± 0.24 cm	1.6 ± 0.18 cm	1.5 ± 0.09 cm	6.0 ± 0.41cm *	198.54	<0.0001
7th	0.8 ± 0.17 cm	2.0 ± 0.16 cm	2.1 ± 0.12 cm	7.4 ± 0.63 cm *	156.77	<0.0001
8th	1.2 ± 0.12 cm	2.5 ± 0.39 cm	2.6 ± 0.33 cm	10.2 ± 0.85 cm *	179.04	<0.0001
9th	1.8 ± 0.16 cm	4.0 ± 0.12 cm	4.2 ± 0.45 cm	14.0 ± 1.12 cm *	213.26	<0.0001
10th	2.5 ± 0.35 cm	5.0 ± 0.28 cm	5.5 ± 0.51 cm	16.0 ± 0.96 cm *	8.36	0.0075

* statistically significant difference (p < 0.0001).

**Table 7:** Effect of number of gellan layers in coating shell on shell thickness after drying, along with germination percentage of coated wheat seeds.

Number of Gellan Layers	0 (Control)	1	2	3	4	5
Shell Thickness, mm	0	0.15 ± 0.03	0.35 ± 0.03	0.55 ± 0.03	0.71 ± 0.04	0.85 ± 0.04
Percentage of germinated pellicle seeds, %	(95–100)	(95–100)	(90–100)	(30–40)	(10–20)	(5–10)
Average seedling length of wheat seeds 5 days after planting, cm	0.2 ± 0.09	3.0 ± 0.2	4.0 ± 0.2	6.0 ± 0.6	4.0 ± 0.5	2.0 ± 0.4
Average seedling length of wheat seeds 10 days after planting	2.5 ± 0.35	10.0 ± 0.4	16.0 ± 0.5	18.0 ± 0.6	14.0 ± 0.5	12.0 ± 0.4

**Table 8:** Growth of uncoated (control) and gel-coated wheat seedlings containing Kaz-6.

Observation Day	5th Day of Observation	10th Day of Observation
Control: Uncoated seeds in the presence of Kaz-6	* 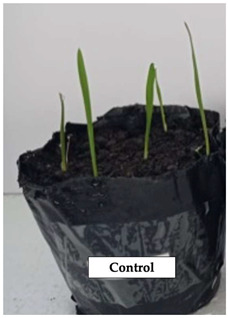 *	* 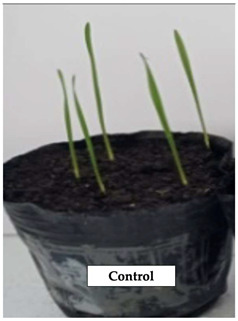 *
Coated with gellan hydrogel and Kaz-6	* 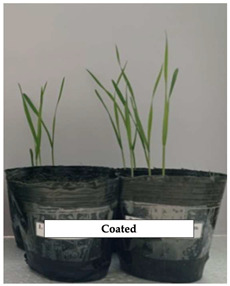 *	* 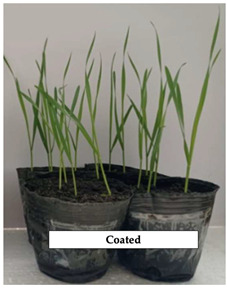 *

## Data Availability

The datasets used and/or analyzed during the present study are available from the corresponding author on reasonable request.
